# Mutation analysis for evaluating code translation

**DOI:** 10.1007/s10664-023-10385-w

**Published:** 2023-12-06

**Authors:** Giovani Guizzo, Jie M. Zhang, Federica Sarro, Christoph Treude, Mark Harman

**Affiliations:** 1 University College London, England, UK; 2 King’s College London, England, UK; 3 University of Melbourne, Melbourne, Australia; 4 Meta Platforms, Inc., Menlo Park, CA USA

**Keywords:** Mutation testing, Source to source translation, Code translation

## Abstract

Source-to-source code translation automatically translates a program from one programming language to another. The existing research on code translation evaluates the effectiveness of their approaches by using either syntactic similarities (e.g., BLEU score), or test execution results. The former does not consider semantics, the latter considers semantics but falls short on the problem of insufficient data and tests. In this paper, we propose **MBTA** (**M**utation-**b**ased Code **T**ranslation **A**nalysis), a novel application of mutation analysis for code translation assessment. We also introduce **MTS** (**M**utation-based **T**ranslation **S**core), a measure to compute the level of trustworthiness of a translator. If a mutant of an input program shows different test execution results from its translated version, the mutant is killed and a translation bug is revealed. Fewer killed mutants indicate better code translation. MBTA is novel in the sense that mutants are compared to their translated counterparts, and not to their original program’s translation. We conduct a proof-of-concept case study with 612 Java-Python program pairs and 75,082 mutants on the code translators TransCoder and j2py to evaluate the feasibility of MBTA. The results reveal that TransCoder and j2py fail to translate 70.44% and 70.64% of the mutants, respectively, i.e., more than two-thirds of all mutants are incorrectly translated by these translators. By analysing the MTS results more closely, we were able to reveal translation bugs not captured by the conventional comparison between the original and translated programs.

## Introduction

Converting a software program from a given programming language into another is often needed by companies that need to migrate their systems to a more recent/popular technology in order to reduce the cost of maintenance in the long run. Furthermore, some companies may require software in a specific programming language in order to seamlessly integrate it into their ecosystem. However, this task poses a series of technical challenges, such as data types conversion, paradigm incompatibilities, floating point precision, and many more (Terekhov and Verhoef 2000) that can rapidly increase the upfront cost, especially when done manually. Terekhov and Verhoef (2000) report in their study what they called “catastrophic failures”: Three companies that went bankrupt, two departments of large technology companies that were dismantled, and an enterprise that lost US$50 million, all because of failed language conversion attempts.

Automating code translation among different languages is thus needed to reduce both the cost and risks of such kinds of projects. Source-to-source translators (also known as transpilers or transcompilers) are tools that can provide this level of automation. These translators can automatically translate source code from one programming language to another (Roziere et al. 2020). Automatic code translation has recently received more attention from researchers, and the state-of-the-art has significantly improved (Aggarwal et al. 2015; Chen et al. 2018b; Karaivanov et al. 2014; Nguyen et al. 2013; Roziere et al. 2020) since its first applications (Waters 1988; Yasumatsu and Doi 1995). Recent advances in code translation include statistical translation (Karaivanov et al. 2014; Nguyen et al. 2013), conventional natural language machine translation techniques (Aggarwal et al. 2015), and deep neural networks (Chen et al. 2018b; Roziere et al. 2020).

One of the most widely used methods of evaluating whether source code is correctly translated to another programming language is the use of Bilingual Evaluation Understudy (BLEU) (Papineni et al. 2002), a measure of syntactic similarities taken from human language machine translation. However, BLEU only evaluates the syntactical correctness of a translation, which is hardly sufficient to assess whether two programs are semantically equivalent. Programs with different syntax might have the same semantics, and different semantics can be derived from similar syntax.

Recently, Roziere et al. (2020) proposed to use test cases to assess the correctness of a translated program in what they called Computational Accuracy (CA). The idea is simple, if a translated program produces the same outputs as the source program using the same set of test cases, then the CA result is high and they deem the translation successful. Although useful for semantic faithfulness assessment, this approach has two shortcomings: First, the assessment is limited to the specific input program itself, which may overfit the training and test data, leading to biased evaluation results of code translators; second, the adopted test suite might be insufficient in exposing the difference between the input program and its translation, yet test suite enhancement remains no easy task (Barr et al. 2015).

Therefore, when using current state-of-the-art techniques, the translation assessment criteria can suffer from various limitations in judging the correctness and trustworthiness of the translated code. An incorrectly translated program assessed as correct may incur human effort to fix the translation bugs that are eventually revealed or, even worse, such bugs may not be revealed in a timely manner. Hence, there is a demand for additional measurements to complement existing ones, to better assess the trustworthiness of code translations.

We propose **Mutation-Based Translation Analysis (MBTA)**, a novel application of mutation analysis for assessing the **trustworthiness** of code translation. A trustworthy translator is expected to capture minor code changes and translate well not only the input program from the test set, but also any other program that is syntactically similar to the input program.

Conventional mutation analysis is used as a method for assessing the test adequacy of a given test suite (Papadakis et al. 2019). A (first-order) mutant is a perturbed program with a small syntactic change generated with a mutation operator. If a test suite produces different outputs between the original program and the mutant, the mutant is said to be killed by the test suite. The mutation score represents the ratio of mutants a test suite kills. A test suite with a higher mutation score has a better ability to reveal synthetic faults, thereby it is hoped it will also prove better at revealing real faults (Papadakis et al. 2018).

Similarly, in MBTA, if a mutant has different outputs from its translation, we deem this mutant as “killed” by the translator. Otherwise, the mutant “survives” the translator. The more mutants a translator kills regarding a program, the less translation adequacy the translator has towards translating this program.

To evaluate MBTA, we perform a proof-of-concept case study with 612 Java-Python program pairs with their respective test cases and a total of 75,082 mutants (which is significantly more than related work (Roziere et al. 2020)). We collect the translation results of two widely-studied translators, namely TransCoder (Roziere et al. 2020) and java2python (j2py) (Melhase 2022). We evaluate the results with our newly proposed MTS measure, as well as with Computational Accuracy (the approach based on test cases proposed by Roziere et al. 2020). We also analyse the relations between specific types of mutants and their translation susceptibility.

The results of our case study show that TransCoder and j2py fail to translate the generated mutants correctly for more than 70% of the cases. Moreover, when analysing the results, we found translation bugs that could not be revealed by using solely the original programs and their translations (and thus only based on Computational Accuracy). In other words, while Computational Accuracy achieved a perfect score for the original translation, MTS highlighted results that were not matching such perfect scores, thus revealing translation bugs. We have also observed interesting scenarios in which the translators successfully translated the original program, but it failed to generate correct translations for *any* of its mutants, thus suggesting overfitting in the translation procedure.

To conclude, the main contributions of this work are:

- We propose Mutation-based Translation Analysis (MBTA), a **novel application of mutation analysis** for code translation assessment, as well as Mutation-based Translation Score (MTS), a **novel code translation measurement** that complements the existing measurements to quantify the trustworthiness of a given translator.
- We conducted **a proof-of-concept case study** with two translators (TransCoder and j2py), 612 programs and 75,082 mutants, and a qualitative analysis of the susceptibility of translators to mutants.
- We provide the code and results of our case study as an open-source online replication package: https://doi.org/10.5522/04/24552178.

## Preliminaries

This section describes the background on code translation (Section 2.1) and conventional mutation analysis (Section 2.2). It also provides a motivating example (Section 2.3) for our work.

### Code Translation

Programming languages keep evolving. Software written in older programming languages needs to be transferred to more recent languages from time to time. However, manual language translation can be error-prone, tedious, and expensive. The Commonwealth Bank of Australia, for example, spent approximately $\$$750 million over five years to switch its platform from COBOL to Java (Roziere et al. 2020).

Code translation tools, or code translators, aim to automatically conduct code translation. Nguyen et al. (2013) and Karaivanov et al. (2014) investigate how well statistical machine translation (SMT) models for natural languages could apply to code translation between Java and C#. Aggarwal et al. (2015) used statistical machine translation models to convert Python 2 code to Python 3 code. These methods are phrase-based, which ignores the grammar information of programming languages. Instead, Chen et al. (2018b) proposed a tree-to-tree-based code translator. Their work is also the first to use deep neural networks toward tackling the code translation problem.

These works rely on the BLEU score to evaluate the translation, which measures the overlap between the tokens in the translation and in the reference. However, the BLEU score does not consider the semantic correctness of the translations, without which the translated code is hardly useful for developers.

Recently, Roziere et al. (2020) proposed an unsupervised approach to automatically translate programming languages. Their approach achieves outstanding effectiveness. Later on, they presented DOBF (Rozière et al. 2021a) and TransCoder-ST (Rozière et al. 2021b), the former pretrains a model to revert the code obfuscation function by training a sequence-to-sequence model; the latter uses automatic test generation techniques to automatically select high-quality translation pairs to fine-tune the pre-trained model. These works use Computational Accuracy (CA), a measure to evaluate the translated code, which is based on the ratio of test cases that have similar outputs between the input program and its translation.

Compared to the BLEU score, CA captures the semantic equivalence of the code translation. Nevertheless, it relies on the fault-revealing ability of available test cases. In addition, an acceptable code translator is expected to translate well not only the input program from the test set, but also any other program that is syntactically similar to the input program. CA only evaluates the translation of a particular input program. It is unclear whether the code translator could still make successful translations once the program is slightly changed if we merely rely on CA assessment.

MTS (presented in Section 3) is a novel mutation-based analysis measurement that complements BLEU score and CA in assessing code translation. Different from BLEU score, MTS captures semantic information of the translated code. Different from CA, MTS provides a more comprehensive assessment on the translation ability of the code translator with multiple mutated input programs. It captures the trustworthiness and robustness of the code translator under evaluation.

 Sun et al. (2020) proposed TransRepair (and later on CAT (Sun et al. 2022)) with semantic-similar word mutation to automatically test machine translation errors. Our work has the following three primary differences with TransRepair: 1) TransRepair focuses on testing translators of text rather than source code. In a sense, the mutation of software shown in our paper is analogous to the mutation of text shown in their work, but they differ drastically in how they are created, evaluated, and analysed. For instance, software mutants need to be executed against test cases to unveil behavioural differences, a property that machine text does not have. The objective of our paper is precisely to showcase how mutation of software and behavioural execution of test cases can be used to test source-code translation. 2) TransRepair conducts context-similar word mutation, while our paper does not have any restriction on the mutation operators in terms of semantic, context, or syntactic similarity. 3) TransRepair has the hypothesis that the unchanged part of the sentence should have a similar translation, but this work does not have such a hypothesis.

### Mutation Analysis

Mutation Analysis is a fault-based criterion to evaluate the testing adequacy of a given test suite (Jia and Harman 2011; Offutt and Untch 2001; Papadakis et al. 2019). In mutation analysis, mutated programs are created by applying mutation operators to the original program. Such mutation operators modify the Software Under Test (SUT) by applying small syntactic changes. A mutant is said to be killed when its output differs from the output of the original program against the SUT’s test suite. Otherwise, the mutant survived. There is a special type of mutant called Equivalent Mutant, which cannot be killed by any possible test suite, i.e., it is semantically equivalent to the original program.

Mutation Testing is the activity of using the results of Mutation Analysis to guide and improve the test suite. In other words, if a (non-equivalent) mutant survives, then the test suite can be enhanced to kill such a mutant. If a test suite kills all mutants, then it is called “adequate”. The level of adequacy of a test suite is measured by the Mutation Score measure, as shown in (1) (Guizzo et al. 2020).

1 $$\begin{aligned} MS(T,M) = \dfrac{DM(T,M)}{|M| - EM(M)} \end{aligned}$$

where *M* is the set of mutants; *T* is the SUT’s set of test cases; *MS*(*T*, *M*) is the mutation score obtained when executing *T* against *M*; *DM*(*T*, *M*) is the number of mutants in *M* killed by *T*; |*M*| is the number of mutants in *M*; and *EM*(*M*) is the number of equivalent mutants in *M*. In summary, the mutation score measures the percentage of non-equivalent mutants killed by the test suite *T*. The greater the mutation score *MS*(*T*, *M*) is, the more adequate *T* is in revealing faults.

### Motivating Example

**Fig. 1:**
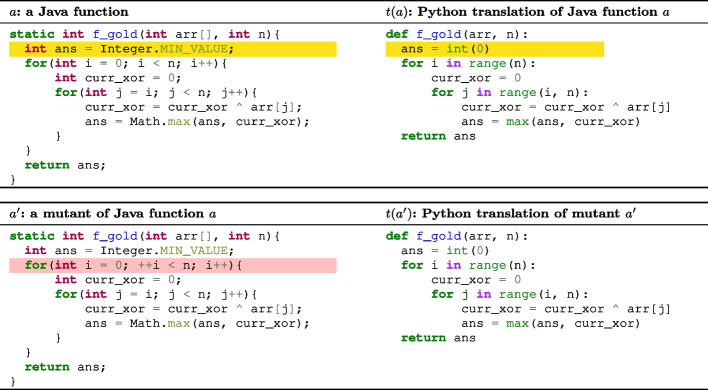
**Motivating Example.** The code translator mistranslates the statement highlighted in yellow. Our mutation analysis exposes this mistranslation that is ignored by test execution. The mutant example also demonstrates that code translators tend to “memorize” coding templates, thereby ignoring the changes in the mutant (highlighted in red) and producing an identical translation to that of the original program

This section presents an example scenario to motivate our approach. Figure 1 depicts a scenario which comes from a real case in our case study. The functionality of the code is to return the maximum XOR subarray value in a given array. The top two code snippets are the original Java function *a* (on the left) and its Python translation *t*(*a*) (on the right) provided by TransCoder. The bottom two snippets are one mutant of the Java function $a'$ (by changing i into ++i in Line 4) and the Python translation of this mutant $t(a')$.

The translation from *a* to *t*(*a*) is incorrect: as the lines that are highlighted in the yellow show, int ans = Integer.MIN_VALUE; is translated into ans = int(0), but the value of Integer.MIN_VALUE is -2147483648 and not 0. However, the available test cases fail to capture the translation bug, given that the minimum value of curr_xor is 0. Consequently, the assessment of this translation produces a false negative.

The mutant we generate ($a'$) changes i into ++i in Line 4, as highlighted in red. The change makes the test input {arr=[33,98],n=1} skip the loop, producing an output of -2147483648, which is different from the translation $t(a')$ with an output of 33. Thus, the mutant is killed during the translation because it produces different test execution outputs before and after translation. This observation exposes the translation bug that the original test execution results are unable to unveil.

In addition, the change from i to ++i is completely ignored by the code translator, which still translates the loop condition into range(n). This suggests that code translators tend to overfit the available data and “memorize” coding templates, thereby ignoring the changes in the mutant and producing an identical translation to that of the original program. This interesting observation also provides another reason to be cautious of the translation of the original program. Even if the original translation is correct, failure in translating mutants with very minor changes (i.e., very similar to the original program) suggests that the code translator may occur in similar bugs in future translations.

## Mutation-Based Translation Analysis

Mutation-Based Translation Analysis (MBTA) provides a criterion to assess the translation trustworthiness of a given source-to-source code translator. Similarly to conventional Mutation Analysis, MBTA uses the mutant execution results to assess a translator. The main difference with conventional Mutation Analysis is that the mutants are instead compared to their own translations, rather than the original program. Thus, a reasonable translator is expected to have a **low** mutation score in code translation, i.e., fewer mutants generating a different output from their translations. That is, the more mutants survive the translator, the better.

**Fig. 2:**
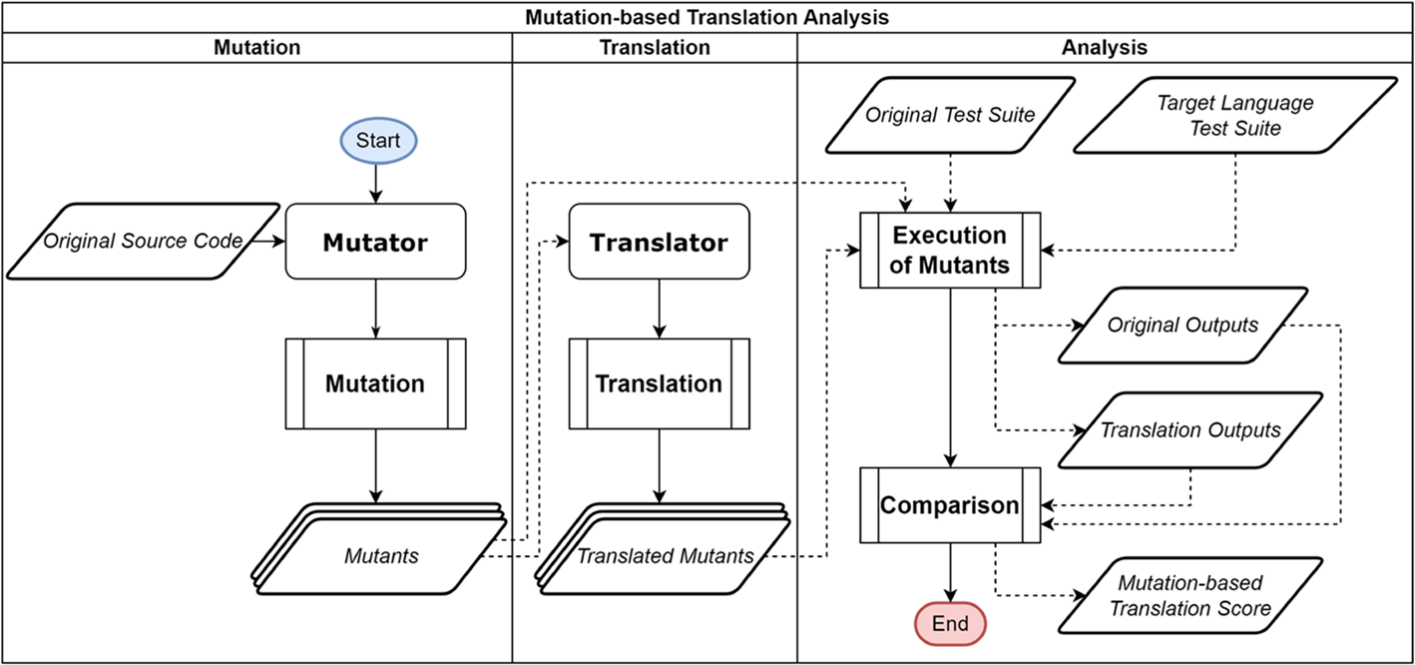
Workflow of Mutation-Based Translation Analysis (MBTA)

Figure 2 presents the workflow of MBTA. MBTA consists of 3 main steps: i) *Mutation*; ii) *Translation*; and iii) *Analysis*. In the first step of *Mutation*, the engineer shall provide the original source code written in the original language. Then a mutator (mutation tool) is used to apply the mutation and generate the mutants for the original program. Such mutants are generated in the same language as the original program.

In the step of *Translation*, the mutants generated in the previous phase are given as input to the translator under test. The translator then translates the mutants from the source language to the target language. The output is a set of translated mutants.

In the final step, *Analysis*, the mutants (both original and translated) are compiled (when necessary) and then executed against their respective test suites. The test suite of the original mutants is the test suite in the original language, usually accompanying the original program. On the other hand, the translated mutants require a test suite in their respective language. The simplest way is for the tester to collect the original test inputs in the source language, and translate them to the target language. The important aspect of this activity is to make sure that both test suites contain equivalent test inputs.

Unlike conventional mutation, the expected output that usually comes with the test cases is not needed for such a process. During the execution of mutants, the oracle that defines the expected output is actually the output of the mutants in the source language. These outputs are the ones that the translated mutants should match, i.e., an original mutant and its respective translated mutant ideally should both produce the same outputs for all inputs. If a translated mutant produces different results than its original mutant, then the translated mutant was incorrectly translated by the translator and the mutant is said to be killed. Therefore, the mutation-based translation analysis can also be regarded as a new form of metamorphic testing (Chen et al. 2018a; Zhang et al. 2014a). Equation (2) presents how to compute whether a mutant is killed:

2 $$\begin{aligned} killed(m|T, m'|T') = \exists t \in T: output(t, m) \ne output(t', m') \end{aligned}$$

where *m* is a mutant in its original language; $m'$ is the respective translated mutant; *T* is the original test suite in the original language; $T'$ is the test suite in the target language; *t* is a test case in *T*; $t'$ is the respective translated test case in $T'$; and *output*(*t*, *m*) is the result of the execution of *t* in *m*.

If a translator can translate all mutants such that none of them mismatches the original mutants’ outputs when executed with the same inputs, then we can increase the trustworthiness of the translator in translating similar programs. However, it is unlikely that a translator will not generate a failing translation for at least one mutant. Hence, in order to evaluate the trustworthiness level of a given translator, we define the **Mutation-based Translation Score (MTS)** in (3):

3 $$\begin{aligned} MTS (M|T, M'|T') = \frac{1}{|M|} \times \sum _{i}^{|M|}{{\left\{ \begin{array}{ll} 1 &{} killed(m_i|T, m'_i|T') \\ 0 &{} otherwise \end{array}\right. }} \end{aligned}$$

where *M* is the set of all available mutants; $M'$ is the respective set of translated mutants; $m_i$ is the *i*-th mutant in *M*; and $m'_i$ is the respective *i*-th translated mutant. In summary, the MTS measure computes the percentage of translated mutants that mismatch their respective original mutant, i.e., the percentage of killed mutants. The greater the MTS, the more translation bugs are revealed. Conversely, the lower the MTS, the better the translator is in generating translations which produce the same result as the original program.

When using MTS with multiple programs, one can analyse it in two ways: i) **Overall MTS** – the percentage of mutants and translated mutants from all programs mismatching their outputs; or ii) **Individual MTS** – the percentage of mismatches per program separately. The former should be used to compute the overall trustworthiness of the translator when considering multiple scenarios, whereas the latter should be used to obtain a more fine-grained result considering specific programs.

Anomalous mutants can be found during the execution of the last step. An anomalous mutant is a mutant that: i) does not compile; ii) generates runtime errors/exceptions; iii) timeout/takes too long to execute (e.g., infinite loops); or iv) does not produce outputs for all the inputs (e.g., kills the process midway). Although such mutants are useful and can be manually analysed by the developer in order to unveil what is causing such anomalies, they are not interesting during the MTS computation due to the inconsistent set of results that can arise. In this case, the default action is to discard such mutants during the MTS computation.

Furthermore, although equivalent mutants are a threat to conventional Mutation Analysis, for MBTA, they do not interfere with the results. This is due to the fact that the outputs of the mutants are used as oracles for the MTS assessment, i.e., they are not compared to the original outputs, but rather with their translations. Therefore, an equivalent mutant does not impact the results of MTS and, if its translation mismatches the original program’s translation, it can further reveal a mismatching behaviour in two translations that should be equivalent.

The engineer can also analyse the results beyond the MTS quantitative values, i.e., the engineer can qualitatively analyse the original program, mutants, and their translations on given occasions. For instance, if a translator fails to translate a program, but successfully translates a mutant, then the difference between the mutant and original program can be analysed to discover the reason why the original translation fails. It can also unveil overfitting if the two translations (original and mutant) are largely different, i.e., the translator loses the ability to generate similar translations for similar programs. Therefore, MBTA can be very helpful for the engineer in revealing new bugs (finding translation bugs in mutants), debugging the cause of failing translations (the translator fails to translate the original program but successfully translates the mutants), and unveiling overfitting (when the original and mutant programs yield largely different translations).

In a broader sense, MTS works similarly to the conventional Computational Accuracy (CA) (Roziere et al. 2020) by measuring whether mutants and their translations match the output of their respective test suites. However, only applying CA to mutants would not be enough, as this would entail issues with the evaluations, including biased CA towards programs with more mutants. This is why we propose the analysis of MTS from many angles, such as MTS per class, MTS per mutation operator, and qualitative analysis of the generated mutants and translations. By doing so, the engineer can have more fine-grained information about the behaviour of their translator.

## Case Study Design

In order to evaluate the MBTA, we design the following research questions:

1. What is the assessment result of MTS on code translators?
2. How does MTS compare with the assessment results of existing criteria?
3. How does MTS assess the code translators with different mutation operators?

### Translators

We use two widely studied code translators that enable the automatic translation from Java to Python in our evaluation.

The first is TransCoder (Roziere et al. 2020), which is an open-source multi-language transpiler that uses self-supervised learning to perform the translations. During training, TransCoder uses a tokenization technique to mask and obfuscate code. Then, it tries to generate a correct completion of the code it just masked and tries to match the original code. During translation, it uses all those learnt tokens and code completions seen during training to provide the translation of unseen programs. It is unsupervised in the sense that it does not require a ground-truth translation, i.e., it works solely with the input programs. We use the pre-trained model provided by Roziere et al. (2020) to translate the set of subject programs written in Java to their respective Python code.^1^

The second is java2python (j2py) (Melhase 2022), an open-source and commercial tool that translates Java code into Python code. j2py, different to TransCoder, is not an ML based technique. Rather, it first translates the code into an Abstract Syntax Tree (AST). Then, j2py walks the tree by generating equivalent nodes in Python using a set of pre-defined translation rules. j2py starts from the assumption that for each Java node, there is one or a set of equivalent nodes in Python. It is a rather simple approach that works well for most cases.

We selected those two translators because they are widely studied for source-to-source translation, are both open-source, require little configuration, and do not require training (TransCoder provides a pre-trained model and j2py is not ML-based), and can be executed in a feasible time. TransCoder is widely adopted in the literature as a baseline for multi-language comparisons (Roziere et al. 2020; Rozière et al. 2021a, b), while java2python is a more practical tool with a specific goal of unidirectional translation. We did not choose DOBF (Rozière et al. 2021a) or TransCoder-ST (Rozière et al. 2021b) in this paper either because they do not support the translation between Java and Python, or because they are not widely studied and used in the literature.

### Subjects

To answer the RQs, we use the same subject programs used by Roziere et al. (2020) in their empirical evaluation of TransCoder. The dataset is composed of 852 programs written for the GeeksforGeeks platform,^2^ an online course website with many challenging problems for which solutions are provided in many languages. The dataset contains 615 Java-Python pairs, from which we used 612^3^ alongside their respective and equivalent test suites (with 10 test cases each). The largest program in the dataset has 127 lines of code and the average program size is 30 lines. These programs and test suites serve as our “ground truth”, i.e., the results of the tests are used as an oracle to decide whether the translations are successful or not.

### Mutation Tool

We use the $\mu$Java (Ma et al. 2005; Offutt 2014) tool to mutate the Java programs. It uses two sets of Mutation Operators: i) class-level operators and ii) method-level operators. The former applies mutations to classes, such as inserting the keyword “super” in a method call or removing the parent’s constructor call. Method-level operators, on the other hand, work on changing the methods’ statements, such as adding an increment to a variable, deleting a line, inverting a conditional operator, and others. $\mu$Java contains 29 class-level operators and 16 method-level operators. We refer the reader to the official $\mu$Java website for a full description of all operators (Offutt 2014).

$\mu$Java offers a comprehensive GUI which allows all tasks involved in mutation analysis. When using $\mu$Java, the engineer first generates mutants at the source-code level and stores them in a dedicated directory. Using $\mu$Java, the engineer can also check the mutations manually and exclude mutants they find equivalent or uninteresting. Then, the engineer can select the test suite and run it against all mutants. In the end, $\mu$Java shows the killed and living mutants so the engineer can enhance their test suite or mark equivalent mutants.

$\mu$Java is a widely used mutation tool in the Mutation Testing literature (Papadakis et al. 2019), which fits our purposes by providing a great gamma of mutation operators and, more importantly, by mutating the source-code, instead of binary code. We have used all of $\mu$Java’s method-level mutation operators in our study. We have not used class-level operators due to the nature of our programs, which do not use any type of class inheritance, and thus not allowing the application of such operators.

### Case Study Procedure

To answer RQ1, we followed the workflow depicted in Fig. 2 and described in Section 3. We report the MTS results and analyse the translation trustworthiness of TransCoder and j2py.

For answering RQ2, we compute the Computational Accuracy (CA) (Roziere et al. 2020) of both translators using the original and translated programs, and then compare the results with MTS. CA measures the percentage of outputs that match a given program and its translated counterpart using the same inputs. Hence, if a program and its translation generate matching outputs for the same input, then CA is incremented. If the program and its translation mismatch their output for a given input, then CA is decremented. In cases where all outputs match, then $CA = 1.0$, whereas $CA = 0.0$ when all outputs mismatch.

RQ3 is answered by analysing the results of MTS with each mutation operator. With this RQ we want to unveil whether different operators generate mutants that are easier/harder to translate, so as to explore what kinds of code translators are weak to translate automatically.

**Fig. 3:**
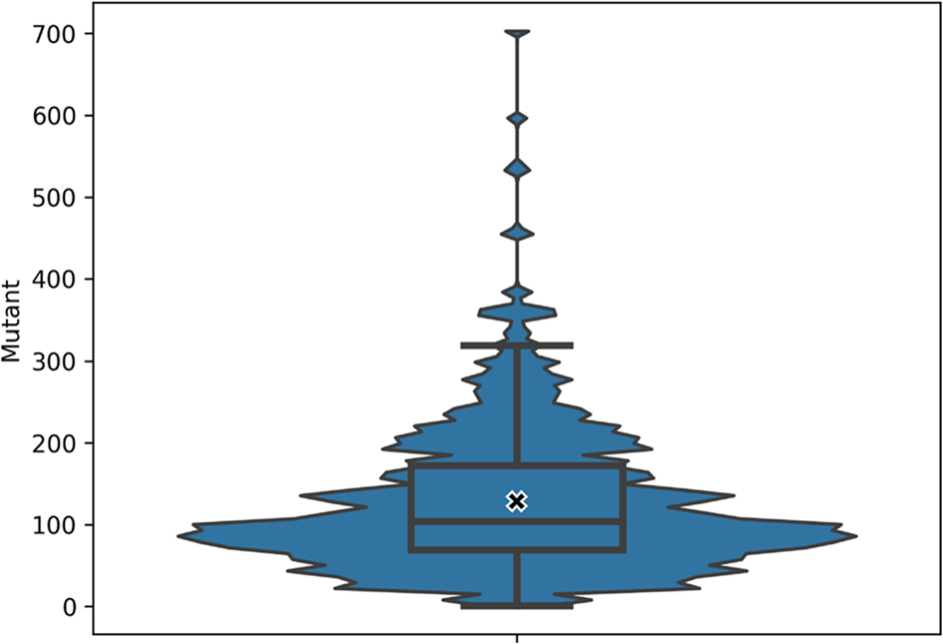
Distribution of the number of generated mutants for Java input programs. The Y axis shows how many mutants were generated for the programs under test. Each data point represents a single program. The “X” marks the mean

## Results

This section summarises the results and provides insights into how they can be used by practitioners to assess the translations of their translators. The programs, mutants, translations, and execution results of our case study can be found in our replication package: https://doi.org/10.5522/04/24552178.

**Fig. 4:**
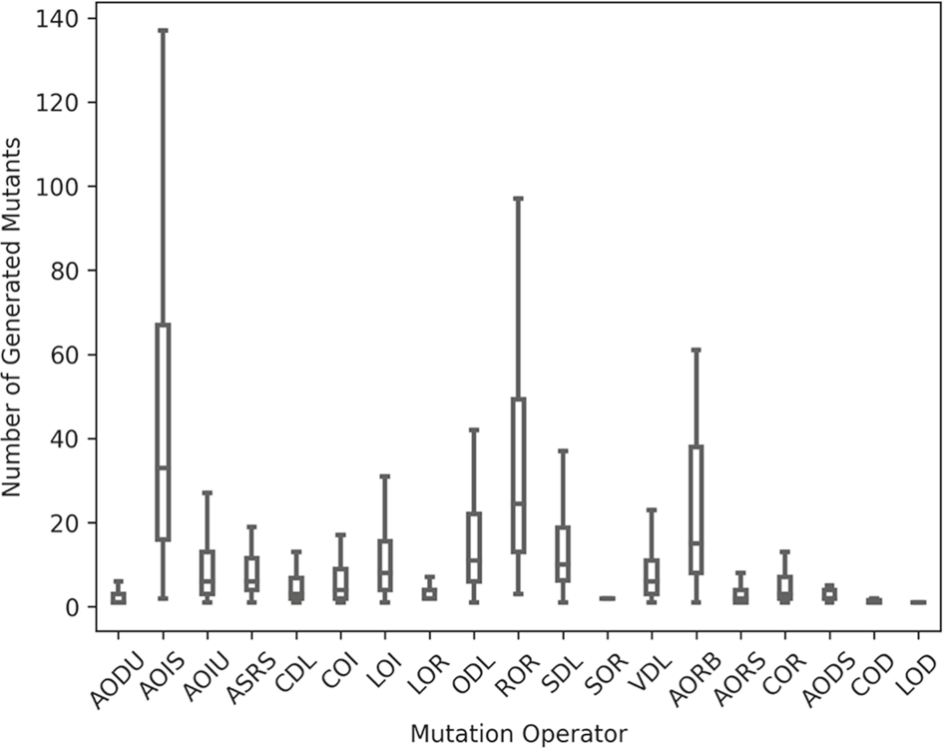
Distribution of the number of generated mutants per mutation operator for Java input programs. The Y-axis depicts the number of generated mutants for the input programs, whereas the X-axis depicts the different mutation operators used in this study. Each data point is one program under test

For the 612 programs considered in our case study, $\mu$Java generated a total of 75,082 mutants. Figure 3 shows the distribution of the number of mutants generated for each program, and Fig. 4 presents the distribution of generated mutants by each mutation operator. Among these 75,082 mutants, 52,386 (69.77%) are non-anomalous, meaning that their executions do not timeout after three seconds with the available test cases,^4^ generate one output for each test input, and do not throw runtime errors. Since we discarded the anomalous mutants, the MTS analysis done hereby considers only the 52,386 non-anomalous mutants and 584 program pairs for which at least one mutant could be used.

### RQ1: MTS Assessment Results

Table 1 presents the general overview of the results for both TransCoder and j2py. TransCoder was able to generate translations for 52,377 (99.98%) of the non-anomalous mutants, out of which 47,729 (91.11%) could be compiled successfully. However, only 26,829 (51.21%) translated mutants are non-anomalous, i.e., could be run successfully (without any runtime errors, or timeouts) and generate one output for each test input. j2py was able to generate translations for 47,247 (90.19%), out of which 13,794 (26.33%) are non-anomalous.

**Fig. 5:**
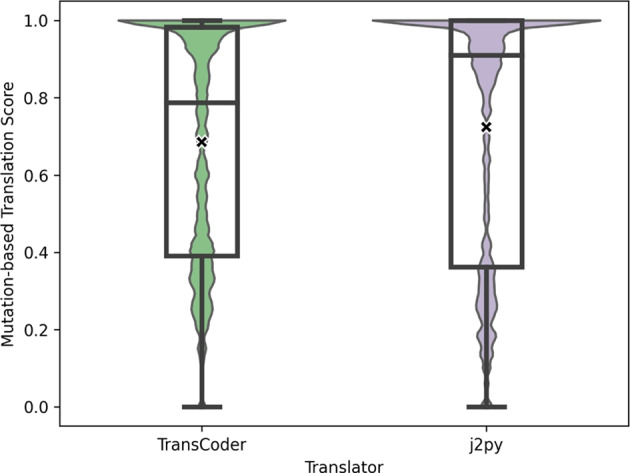
RQ1-2: Distribution of the MTS (lower is better) per program. The Y-axis depicts the MTS for each program under test, whereas the X-axis depicts the two translators used in this study. Each data point represents the overall MTS of a single program under test. The “X” marks the mean

Out of the original 52,386 non-anomalous mutants, 36,899 (70.44%) translated mutants by TransCoder obtained at least one different output as their respective translation, i.e., the overall MTS of our case study is 0.7044. Similarly, the MTS of j2py translations is 0.7064, meaning that j2py also failed to translate more than two-thirds of the mutants. Figure 5 presents the distribution of the MTS results for the programs considered in this paper using TransCoder and j2py.

**Table 1:** RQ1-2: Results for mutant generation (top row), overall MTS (middle row, lower is better), and Computational Accuracy (CA, bottom row, higher is better)

Measure	TransCoder	j2py
Non-anomalous mutants	52,386	52,386
Translation outputs	52,377 (99.98%)	47,247 (90.19%)
Compilable translations	47,729 (91.11%)	39,237 (74.90%)
Timeouts	496 (0.95%)	883 (1.69%)
Exceptions	19,018 (36.30%)	23,859 (45.54%)
Non-anomalous translations	26,829 (51.21%)	13,794 (26.33%)
Overall MTS	0.7044	0.7064
Median Individual MTS	0.7877	0.9101
Mean Individual MTS	0.6848	0.7235
Std. Dev. Individual MTS	0.3034	0.3304
Overall CA	0.3632	0.3279
Median Individual CA	0.0	0.0
Mean Individual CA	0.3700	0.3279
Std. Dev. Individual CA	0.4545	0.4566

The percentages in the first two rows are calculated against the total number of non-anomalous mutants. CA is calculated against the number of original programs

As Fig. 5 shows, the most frequent MTS result is 1.0, i.e., all of the mutants for that program entirely mismatch the output of their translated counterparts. In fact, for the TransCoder results, 109 programs (18.66%) achieve an MTS of 1.0 (full mismatch), and only six programs (1.03%) achieve an MTS of 0.0 (full match). However, all six programs with an MTS of 0.0 have less than 10 mutants each, whereas only three of the 109 programs with an MTS of 1.0 have less than 10 mutants. For j2py, we obtained an MTS of 1.0 for 151 programs (26.88%) and 0.0 for 9 programs (1.54%).

In summary, by using MTS we discovered that more than two-thirds of all mutants were incorrectly translated using TransCoder and j2py. Furthermore, for almost a fifth of all programs, TransCoder did not generate a single correct mutant translation, whereas j2py failed to translate a mutant for more than a quarter of all programs. Strictly speaking, according to our MTS results, TransCoder is a more trustworthy translator due to the lower number of failing mutant translations. It is worth noting that both translators do not always fail on the same input programs (although they do sometimes fail on the same inputs). We hypothesise that this is due to the different strategies used by both translators: TransCoder uses ML and j2py uses a set of pre-defined transformation rules applied on a syntax tree traversal.

### RQ2: MTS vs Computational Accuracy

**Fig. 6:**
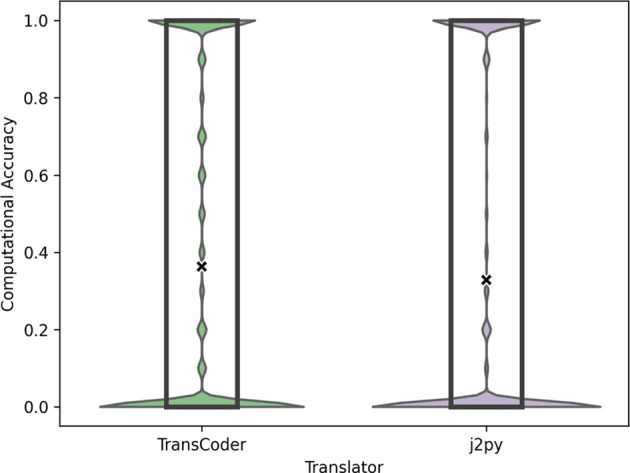
RQ2: Distribution of the Computational Accuracy (higher is better) per program. The Y-axis depicts the CA for each program under test, whereas the X-axis depicts the two translators used in this study. Each data point represents the overall CA of a single program under test. The “X” marks the mean

Recapitulating, the Computational Accuracy (CA) (Roziere et al. 2020) measures the percentage of test inputs of a given program that generate the same output as the translated program, i.e., it quantifies the level of accuracy of a translated program when exercised with the same inputs used with the original program. The CA obtained by TransCoder and j2py in our case study when considering all test cases is 0.3632 and 0.3279, respectively. In other words, for all test inputs, both the original and translated programs achieve the same output in 36.32% and 32.79% of the cases for the respective translators. Figure 6 depicts the distribution of the CA results of the programs for both TransCoder and j2py.

As evidenced by these results, most programs either produce a CA of 0 (no output match) or 1 (all outputs match), skewing the data towards both boundaries. In fact, for the TransCoder results, for 55.14% of the programs the CA is 0 (incorrect outputs for all inputs), for 29.62% of the cases it is 1 (adequate translation), and for 70.38% of the cases it is lower than 1 (faulty translation with at least one incorrect output). Similarly, j2py obtains a CA of 0 for 62.96% of the programs, 1 for 29.43% of the programs, and lower than 1 for 70.57% of the cases.

When comparing MTS to CA, we confirmed what we expected for their correlation. CA and MTS are strongly and inversely correlated according to Spearman’s $\rho$ test -0.78 (p-value < 0.001), meaning that they tend to agree with each other inversely. This inverse correlation was expected because they are measuring quality in different directions: higher CA and lower MTS are better for the translator. They are correlated because, as we previously expected, if the translator can successfully translate the original program, it is very likely that it will also successfully translate similar variations of this program.

We also found that MTS reveals approximately the same proportion of bugs as CA. For example, when using MTS and CA to evaluate TransCoder, the metrics are able to reveal that 70.44% and 70.38% of the translations are faulty, respectively. Similarly, for j2py, MTS reveals a ratio of 70.64% of faulty translations, whereas CA reveals a faulty translation proportion of 70.57%. This evidences even further that both metrics tend to agree on their results. Upon further analysing the results, we found out that when the CA of a program translation is 1 (all test inputs pass on the translation), the MTS result tends to be low with a median of $\approx$0.3, i.e., 30% of the mutants die and 70% of the mutants pass the translation check when the original program is adequately translated. On the other hand, when the CA of a program is 0 (incorrect outputs for all inputs), then MTS tends to be close to 1.0 with a median of 0.98, i.e., approximately 98% of mutants fail to be translated when the original program is incorrectly translated. Measuring MTS on programs that obtain 0 CA can reveal cases where a given translated mutant passes the test suite and the engineer can check what is causing the original program translation to fail. These types of mismatches between the two metrics provide useful information to the engineer.

MTS also provides a more fine-grained measurement with a different distribution of values. This difference in distributions is evident in Figs. 5 and 6, where the MTS values are more dispersed along the Y axis, thus enabling a more informative analysis. Moreover, if we look at the total number of bugs revealed in the TransCoder translations, due to the greater amount of mutants than programs, MTS can naturally reveal more bugs (36,899 mutants versus 411 original programs failing the translation). This can be extremely useful for an engineer assessing a translator, since they are given more cases in which their translator can fail. For example, by analysing the results of MBTA on the original program with the following statement “while ((int) (x & m)>= 1)”, we found that TransCoder translates the following mutated Java statement “while ((int) (--x & m)>= 1)” into the Python statement “while int((--x & m)>= 1)”.^5^ It may seem correct at first, but it neglects the fact that “--” preceding a number variable in Python is simply interpreted as a double sign negation, thus rendering the translated mutant equivalent to the original translated program and different to the mutant. The correct translation would be to keep the original statement “while int((x & m)>= 1)”, while also including the assignment $x = x - 1$ before exiting the loop. This translation bug could only be revealed with MBTA, since none of the 612 original Java programs uses the operator “--” before a variable in an expression evaluation.

Although TransCoder obtains a perfect CA (1) with the original program of the aforementioned example, the MTS for the same program is 0.4531. In other words, small syntactic changes to that program will make TransCoder generate a faulty translation in almost half of the cases. This phenomenon was also observed in other programs. For 168 programs, TransCoder obtains a perfect CA, but the MTS results are greater than 0, i.e., the original translation seems correct, but the tool fails to translate at least one of its mutants. j2py fails to translate mutants for 147 programs for which it obtains a perfect CA.

Another interesting phenomenon occurred with a program for which the CA is 1, but the MTS is also 1. In other words, TransCoder generated a correct translation (which we manually checked) for the original program, but it failed to translate any mutant of those programs. In this case, it seems that TransCoder is overfitting to the original program, and any syntactic change leads to a wrong translation. In such situations, the engineer may be compelled to include programs with small variations during the training process in order to generate models capable of translating programs similar to the ones represented by the generated mutants.

We also found one case where CA is 0 (the original translation is incorrect on all test cases) and MTS is also 0 (all mutant translations pass all test cases). In such case, only six mutants were generated for that program, which means that only six mutants need to pass the test cases to obtain an MTS of 0 and the original program must fail the 10 test cases to obtain a CA of 0. With such a low number of mutants, this may occasionally happen, although such a scenario is unlikely. In general, as shown by our case study, we do not expect the translators to incorrectly translate the original program and correctly translate potentially hundreds of similar mutants as both metrics tend to agree.

We have also performed a statistical analysis on the correlations between the number of generated mutants for a given program and the obtained MTS and CA using Spearman’s $\rho$ test (Spearman 1904). The intuition is that, as the number of mutants increases, one could expect the trustworthiness measures to also increase or decrease. However, we have only found negligible to very weak correlations between the number of mutants and either CA or MTS, i.e., correlations with Spearman’s $\rho$ test returned 0.16 and 0.22 respectively (p-value < 0.001). Therefore, the number of mutants is not a good predictor for the obtained CA and MTS of a program.

All in all, we can state that MTS is especially useful for unveiling bugs since it provides more programs to test the translators, as opposed to using only one with CA. With MTS, the engineer can look at more fine-grained and complementary information to unveil specific cases in which their translators fail. Hence, we believe MTS can complement the CA measure by providing additional information on the trustworthiness of the translations and insights on possible overfittings.

### RQ3: Results of each Mutation Operator

Table 2 presents the MTS for each of the 19 $\mu$Java’s mutation-level operators (Ma et al. 2005; Offutt 2014) over 584 programs. We refer the reader to $\mu$Java’s website for the complete description of all mutation operators (Offutt 2014).

**Table 2:** RQ3: MTS by $\mu$Java’s mutation operators

Operator	Description	TransCoder MTS	j2py MTS
AODS	Arithmetic Operator Deletion (short-cut)	0.85	1.00
AODU	Arithmetic Operator Deletion (unary)	0.52	0.67
AOIS	Arithmetic Operator Insertion (short-cut)	0.90	0.91
AOIU	Arithmetic Operator Insertion (unary)	0.58	0.71
AORB	Arithmetic Operator Replacement (binary)	0.67	0.74
AORS	Arithmetic Operator Replacement (short-cut)	0.63	0.81
ASRS	Assignment Operator Replacement (short-cut)	0.69	0.80
CDL	Constant Deletion	0.67	0.77
COD	Conditional Operator Deletion	0.67	0.86
COI	Conditional Operator Insertion	0.63	0.68
COR	Conditional Operator Replacement	0.75	0.81
LOD	Logical Operator Deletion	0.50	0.50
LOI	Logical Operator Insertion	0.61	0.71
LOR	Logical Operator Replacement	0.42	0.35
ODL	Operator Deletion	0.66	0.74
ROR	Relational Operator Replacement	0.62	0.72
SDL	Statement Deletion	0.59	0.71
SOR	Shift Operator Replacement	0.54	0.69
VDL	Variable Deletion	0.67	0.77
Median		0.63	0.74
Mean		0.64	0.74
Standard Deviation		0.11	0.14

Interestingly enough, one of the simplest mutation operators, the Insert arithmetic short-cut operator (AOIS), is the one with the highest MTS for both translators. This operator is designed to insert “$++$” and “$--$” before or after variables in expressions. While in Java they are used to increase or decrease numerical values, in Python they perform a double change in signal for the variables they precede. Such operations are correctly translated by TransCoder when appearing in a for loop (e.g., Java’s “for(int i = 0; i < 10; i++)” is correctly translated to Python’s “for i in range(0, 10):”), but are incorrectly translated in situations where the incremented variable is being used in the same statement (e.g., in the example given in the previous subsection). This high MTS is probably due to the fact that the programs used in the training process of TransCoder do not contain variables being incremented or decremented while being used in the same expression. Hence, the model is not trained to deal with such situations. Moreover, j2py seems to also fail to recognise this different behaviour between both programming languages by placing the increment/decrement before the loop.

The Logical Operation Replacement (LOR) operator obtains the best MTS. These mutations switch logical operators (e.g., Java’s “ &&” and “||”) with other logical operations. This is most of the time a simple mutation with a straightforward translation from one language to another. Moreover, such logical operations are very common in any program, including the ones used to train the TransCoder model used in this case study.

With these results in mind, we can state that the type of mutation plays an important role in the translation results of both TransCoder and j2py. Finally, by analysing the information unveiled by the MTS results, the engineer can focus on improving certain aspects of their translators. For instance, in the case of the case study presented in this paper, we discovered that both TransCoder and j2py should be improved in regard to short-cut arithmetic operations.

### Threats to Validity

#### Threats to External Validity

As it happens with most Software Engineering papers, the set of program subjects may not be representative of the overall population of software, thus the generalisation of our results may not hold with different datasets. To mitigate this threat, we collected a dataset with 612 programs performing multiple computational tasks, of different sizes, and with a ground-truth version in the target programming language. We have used two translators in our evaluation. Using other translators could produce different results and conclusions. In order to cater for this threat, we specifically selected a well-known translator in the literature and an open-source commercial translator, both using different translation techniques.

#### Threats to Internal Validity

We used a pre-trained model with TransCoder in order to generate the translations. We decided to use such a model for a fair comparison with the results of Roziere et al. (2020). One possible threat is obtaining different results with a different model, generated with different hyper-parameters during training.

#### Threats to Construct Validity

Similarly to the conventional Mutation Score in Mutation Analysis, MTS could be inflated by redundant and easy-to-kill mutants. This could induce a low level of trustworthiness for the translators. However, in our case study, we observed that even small changes to the source code often result in different translations. In this sense, even though similar mutants may be redundant in the original programming language, they may differ when translated.

Another threat to construct validity may be related to the strength of the test suites. A weak test suite may achieve a high CA thus leading to incorrect results regarding this measure. However, although it is a threat to the CA results presented in this paper, this is precisely where MBTA shines. As shown by our results, it is very unlikely that a test-passing translation of a given program will have all of its translated mutants also passing the tests. With that in mind, the engineer can analyse the MTS results and pinpoint translations of the original program that might be incorrect, despite having perfectly matching test outputs.

## Discussion

In this section, we discuss the advantages and limitations of using MBTA, and the main differences between robustness and trustworthiness.

### Advantages and Limitations of MBTA

MBTA has the following advantages:

#### MBTA provides a larger gamma of “test cases”

In other words, instead of using a single original program to check whether a translator is capable of producing correct translations, with MBTA, an engineer can generate multiple program variants in order to test the translator. This can also be used to automatically enhance datasets by providing more valid programs, which can be paired with their test-equivalent translations.

#### No need for a pre-defined output oracle

With MBTA, there is no need for an oracle to define expected outputs. The expected outputs can be derived from the results of the original mutants.

#### MBTA checks for trustworthiness

Whereas common measures such as BLEU check whether a translator is able to syntactically match a piece of code, MTBA goes beyond and checks how good a translator is when faced with unseen programs. Much like mutation analysis checks whether a test suite is adequate to find bugs, MBTA tests whether a translator is adequate to find good translations.

#### MBTA can help find more fine-grained translations

Since MBTA uses mutation analysis as part of its process, the “test cases” (mutants) represent only small syntactic changes, thus we can expect that the translator shall perform similarly well/badly. Indeed, if a translator is able to adequately translate a piece of code, it is reasonable to expect that the translator will produce adequate translations of a similar program with a small syntactic change. If this is not the case, then the engineer can further analyse the results, discover why the translator failed to translate such a similar program, and consequently improve the translator. With this fine-grained analysis, the engineer can tackle specific weaknesses of their translator in unseen programs and improve it.

#### MBTA does not need many translations of test suites

When the engineer needs to test their translator against many programs, then as many test suites written in the target language are necessary to test for correctness. Translating test suites is still a manual task that is error-prone and requires considerable effort from the engineer. With MBTA, instead of testing the translator against many programs, the engineer can translate the mutants of a single program and use a single test suite for all of them. Not only is it easier to use one program, but also the cost of searching for test suites in the target language, or even translating them, is considerably reduced.

#### Equivalent mutants is not an issue

Since the results of the mutants and their translations are not compared to the original programs, equivalent mutants play no role in the MTS results. In fact, they can be powerful assets during the analysis. Because one can expect a mutant’s translation to be equivalent to the original translation, if the mutant is killed and the original translation passes the test, the engineer can unveil a clear translation bug.

The authors of this paper have explored many novel applications of mutation analysis, such as using mutation for providing baselines for fairness improvement methods (Hort et al. 2021), assessing machine learning model redundancy (Zhang et al. 2023), increasing test coverage (Zhang et al. 2016a), and detecting bugs via program behaviour deduction (Zhang et al. 2018). In this paper, we explored how mutation analysis works on source-to-source code translation. In theory, mutation analysis can also be applied to assess other types of code translation tasks, such as code generation (Sun et al. 2020), code summary generation (Chen and Zhou 2018), code comment generation (Hu et al. 2018), and others.

MBTA also has some limitations, as described below:

#### MBTA is sensible to the mutation space and tool

A smaller set of mutants would reduce the ability of MTS in revealing translation faults, but it would not affect its soundness. Analysing all the possible mutants for a program is nearly (if not) impossible due to the potentially infinite number of variants a program can have. For situations where efficiency is highly concerned, we recommend engineers to consider using mutation cost reduction techniques such as selective mutation testing (Zhang et al. 2014b; Papadakis et al. 2019) and predictive mutation testing (Zhang et al. 2016b).

#### MBTA can inflate when redundant tests and mutants are considered

In situations where redundant test cases (i.e., test cases that test the same behaviour) and easily killable mutants are considered, much as it happens to conventional mutation score, MTS may lead to an inflated result. In other words, the translator may seem like close to fully adequate, when in reality it may be only translating a set of “easy to translate” mutants or using many test cases that test the same behaviour.

#### MBTA is sensible to the quality of test suite

If a weak or small test suite is used, it is likely that the original and translated mutants are not tested enough to unveil errors in the translation. This is a limitation of mutation analysis in general as well: the weaker the test suite, the fewer mutations it can kill.

#### Covering mutated programs does not necessarily mean killing mutants

Because the programs we used in our experiments are generally simple, most programs are covered in their entirety (in terms of statement coverage). Unfortunately, covering a mutated statement does not necessarily mean that the mutant will be revealed, thus impacting MBTA. However, if an original mutant is not covered by the test cases and it is killed during MBTA, it means that the mutation failed, either because the translated mutant is covered by the same test cases or because there is a translation bug in one of the covered statements. In this case, the mutation triggered a translation bug, which can be evaluated by the engineer to improve the translation effectiveness.

#### MBTA needs a test suite in the target language

The dataset used in our case study contains equivalent test cases for both languages. However, one cannot assume this is true for all programs. This is indeed a limitation of MBTA: it requires equivalent test cases. However, depending on the complexity of test cases, they can be automatically translated to other languages by translating the inputs with simple scripts. If manual translation is required, a subset of test cases can be used instead, but it would hinder the revealing of translation bugs.

### Trustworthiness and Robustness

This section discusses the main differences between trustworthiness and robustness in translation systems, because they may sound similar and both involve input changes, although we are not aware of any existing work on either of them.

“Robustness” (Zhang et al. 2020) is the resilience of the translator when faced with input perturbations, i.e., refers to the degree to which a system continues to function in the presence of invalid inputs. In our work, if the translator can generate the exact same output with and without perturbation, then the translator is said to be robust.

“Trustworthiness” is a measure of translation quality, rather than model robustness. In other words, if we treat the translator as a black box and analyse only the input-output pair, a translator is said to be trustworthy when it can correctly generate translations for all perturbations, regardless of whether the perturbed program is semantically equivalent to the original or not. Hence, trustworthiness is the ability of a code translator to produce reliable code translations whenever required, including mutants that are valid inputs to the code translator.

In fact, the two concepts of trustworthiness and robustness are closely related and may seem to overlap, but there is a key distinction that separates them as summarised below: 1) the inputs to study robustness are mostly (but not always) invalid; 2) for an ML system with good robustness, its outputs are expected to remain the same despite the changes in the inputs.

For example, to test translation robustness, one can generate the adversarial example ‘x += 2’ for the Java statement ‘x += 1’, both with similar (but not the same) behaviour. If the translator can generate the same correct output in Python ‘x += 1’ in both cases, then we can say the translator is robust enough to translate two semantically similar statements. If it fails, then the translator is not robust to adversarial attacks. In the case of trustworthiness, the translator would be required to provide the correct translation ‘x += 2’ that generates different outputs from the original one. If the translator generates the same Python translation for original and mutated cases, it means that the translator does not provide trustworthy translations to slightly different programs.

The two concepts differ because a trustworthy translator is the one that generates the correct translation of the perturbation, rather than neglecting it and generating the original translation. In other words, if a translator is robust yet it ignores the syntactic and semantic changes to the input and generates the original program’s translation, it is not trustworthy because it cannot generate the correct translation to a different program.

A key aspect of MBTA is that it considers all compiling perturbations as valid alternative programs rather than “noise” or incorrect inputs. Hence, a mutant is not an adversarial attack, but rather another program. Therefore, our approach measures trustworthiness because MTS measures precisely how many perturbations (valid but similar programs) are correctly translated and not how many perturbations are ignored.

## Conclusion

In this paper, we propose MBTA, a mutation-based technique to evaluate the trustworthiness of translators. MBTA uses the concepts of mutants from Mutation Analysis, but it does it differently. Instead of comparing the outputs of a given mutant with the outputs of the original program, in MBTA we compare the mutant outputs with its translation outputs. When such outputs mismatch, then a translation bug is revealed. We also propose the MTS measure, which quantifies the level of trustworthiness of a given translator.

We perform a case study evaluation of MTS using TransCoder, a well-known and recent translator in the literature, and j2python, an open-source commercial translator, over a total of 612 programs and 75,082 mutants. Our results show that these translators fail to translate more than two-thirds of all mutants. Moreover, by analysing the mutants killed, we were able to reveal bugs that could not be captured using measures based solely on the original programs and their translations, such as the CA measure. Finally, we discovered that some types of mutations are more likely to produce incorrect translations, unveiling possible weaknesses in the translators. As shown in our examples, MTS is able to reveal bugs even when the original program and its translation produce the same outputs (i.e., the CA score is perfect). In such cases, MTS can either reveal a translation bug in an unseen program (mutant) or a missed bug in the original translation due to a weak test suite.

For future work, we intend to compare additional translators for different programming languages in order to evaluate whether MTS can be helpful in different scenarios. We also intend to perform experiments with other datasets and mutation tools. Another possibility is to use the mutation results to aid the fault localisation tasks in the context of source-to-source translation. By comparing the mutant translation code to the original translation code, we can pinpoint cases where the original translation passes the test suite but the mutant translation is killed, hence potentially revealing faulty lines of code by analysing the diff between them.

## Data Availability

The datasets generated during and/or analysed during the current study are available in the UCL’s repository, https://doi.org/10.5522/04/24552178

## Footnotes

1 https://dl.fbaipublicfiles.com/transcoder/pre_trained_models/TransCoder_model_1.pth

2 https://practice.geeksforgeeks.org/

3 We discarded three programs because they were raising exceptions during execution.

4 The three seconds threshold is set to capture infinite loops or behaviour that makes the program hang. All the original programs take less than 0.5 seconds to run.

5 The pre-decrement operator -- in C, C++, and Java evaluates to x-1 and decrements the value of *x*.
